# Outcomes and Hand Use of Reaching Attempts: Comparison of Infants at Risk for Developmental Disability and Infants With Typical Development

**DOI:** 10.3389/fpsyg.2022.712252

**Published:** 2022-06-03

**Authors:** Nushka Remec, Judy Zhou, Joanne Shida-Tokeshi, Trevor A. Pickering, Douglas L. Vanderbilt, Beth A. Smith

**Affiliations:** ^1^Neuroscience Graduate Program, University of Southern California, Los Angeles, CA, United States; ^2^Division of Biokinesiology and Physical Therapy, University of Southern California, Los Angeles, CA, United States; ^3^Department of Population and Public Health Sciences, Keck School of Medicine, University of Southern California, Los Angeles, CA, United States; ^4^Section of Developmental-Behavioral Pediatrics, Keck School of Medicine, University of Southern California, Los Angeles, CA, United States; ^5^Department of Pediatrics, Keck School of Medicine, University of Southern California, Los Angeles, CA, United States; ^6^Division of Research on Children, Youth, and Families, Children’s Hospital Los Angeles, Los Angeles, CA, United States; ^7^Developmental Neuroscience and Neurogenetics Program, The Saban Research Institute, Los Angeles, CA, United States

**Keywords:** infant, reaching, developmental disability, grasp, hand use

## Abstract

**Background:**

Infants at risk for developmental disabilities often show signs of motor delay. Reaching is a skill that can help us identify atypical motor trajectories in early infancy. Researchers have studied performance after onset of reaching, but none have followed infants at risk from pre-reaching to skilled reaching.

**Aims:**

We assessed differences in reaching outcomes and hand use as reaching skill emerged in infants at risk for developmental disabilities and with typical development.

**Methods and Procedures:**

We followed infants at risk for developmental disabilities (*n* = 11) and infants with typical development (*n* = 21) longitudinally as they developed reaching skill. Infants reached for a toy at midline while sitting in the caregiver’s lap. Video data were coded for reach outcome (miss, touch, partial grasp, and whole-hand grasp) and hand use (right, left, and bilateral).

**Outcomes and Results:**

Infants at risk had a larger proportion of missed reaches across visits compared to infants with typical development. Infants at risk also showed less variability in hand use when grasping over the study period.

**Conclusion and Implications:**

Our results provide information to support early differences in reaching performance to inform identification of typical and atypical developmental trajectories. Future studies should assess how the missed reaches are different and consider other quantitative measures of movement variability in infants at risk.

## Introduction

Infants at risk for developmental disabilities (AR) make up a diverse group that require careful monitoring of developmental milestone achievement (e.g., the motor milestone of reaching onset). Infants born preterm are considered at risk for poor neurodevelopmental outcomes, as are infants with low birth weight, signs of systemic illness, abnormal metabolism, or congenital impairments (Raju, 2012). Typically, these infants are cared for in the neonatal intensive care unit and are discharged with follow-up for 18–24 months. Follow-up visits typically include assessment of motor milestones using the Alberta Infant Motor Scale or Bayley Scales of Infant Development (Novak and Morgan, 2019). While these observational scales provide a general overview of motor skill attainment, they were not designed to provide a detailed, quantitative assessment of the development of one particular skill, such as reaching.

Reaching typically emerges around 3–5 months of age in infants with typical development (TD; von Hofsten, 1991; Thelen et al., 1993). Prior to reaching, hand shape shifts from a fisted to a semi-flexed position (Sacrey and Whishaw, 2010). To compare reaching behavior in infants AR to infants with TD, researchers have measured kinematics, object exploration, and hand use. For example, infants born preterm have significantly more movement units, less straightness in their reaches, and more inconsistency with hand use compared to infants with TD (Ronnqvist and Domellof, 2006). Infants born preterm have less frequent object contacts, decreased contact duration (Heathcock et al., 2008), more bilateral reaches, and more reaches that result in touch than a grasp compared to infants with TD (de Almeida Soares et al., 2014). Differences in reaching have also been observed in infants with established diagnoses associated with atypical development. Infants with cerebral palsy have longer reach durations (Boxum et al., 2017) and reach less frequently and slower (Mazzarella et al., 2020) than infants with TD. Infants with Down syndrome have fewer successful reaches in a 1-min period than infants with TD (de Campos et al., 2013). Infants with perinatal stroke reach for toys less often and touch toys for shorter durations when using their affected vs. unaffected side (Chen et al., 2015). Ouss et al. (2018) found that infants with West syndrome have decreased hand curvature compared with infants with TD when reaching to a toy.

Handedness is thought to be indicative of hemispheric specialization of function (Michel et al., 2013). A review by Michel and colleagues describes studies in which early establishment of hand preferences in infants with TD (maintained across longitudinal assessments) predicts advances in language development, tool use, and objects construction skill (Michel et al., 2016). However, flexibility in hand preference over time is also observed in infants with TD (Thelen et al., 1993). The operational definition of consistency and the balance between consistency and flexibility of behavior is important to consider when we compare infants AR to infants with TD. In infants with or at risk of atypical development, a very strong consistent hand use preference is likely indicative of underlying neuromotor pathway impairment. Grasp, reach, and hand use and preference are important features in motor development and have the potential to inform us about underlying neuromotor function.

Previous studies on infant reaching have not considered movement variability in infants AR. We define movement variability as shifts between unilateral vs. bilateral and right vs. left arm attempts during reaching as this skill emerges over time. How many unilateral vs. bilateral reach attempts infants make, how many attempts they make using their right vs. left arms, and how they vary within and across assessments may influence the learning process. Movement variability has been studied in adult reaching (Wu et al., 2014) and infant postural control (Dusing et al., 2014), suggesting that low amounts of variability are atypical and could indicate difficulties with flexibility in different movement contexts. Analyzing variability in unilateral vs. bilateral and right vs. left reaching attempts within and across assessments is an important first step in assessing if infants AR are impaired in movement exploration when learning a new skill.

The current literature is also limited by a lack of longitudinal data from pre-reaching to skilled reaching in infants AR. Most studies follow infants longitudinally after reaching onset up to about six or 7 months of age (Fallang et al., 2003; Toledo and Tudella, 2008; de Almeida Soares et al., 2014) and are thus missing the pre-reaching period. Thelen et al. (1993) defined the point of reach onset as the first week in which infants consistently contact an object by flexing the shoulder and extending the elbow while maintaining visual gaze on the object. Others have defined it as the point of three to five object touches in 1 min (de Almeida Soares et al., 2014) or the week at which number of object contacts exceeded three times the previous week (Bhat and Galloway, 2006). To date, studies including pre-reaching attempts have only included infants with TD (Corbetta and Thelen, 1999; Bhat and Galloway, 2006) or were cross-sectional studies (Mazzarella et al., 2020).

Given the lack of longitudinal studies measuring the outcomes and hand use of infant reaching attempts from pre-reaching to skilled reaching in infants at risk, our purpose here was to assess reaching performance of infants AR and with TD across the time period of reaching skill development. First, we described the infants’ developmental trajectory according to the Bayley Scales of Infant Development, 3rd edition subscales. Then, we compared the number of total reach attempts, proportion of different reach outcomes, and changes in unilateral and right-sided reaching attempts. Lastly, we assessed the variability of hand use for grasping and contacting objects across visits. Reach outcomes were classified as unsuccessful or successful—if unsuccessful, were categorized as a “miss,” and, if successful, were categorized as either a touch, partial-hand grasp, or whole-hand grasp. Our results describe early differences in reaching performance between infants AR and with TD as reaching skill emerges, inform the early identification of atypical developmental trajectories, and identify potential targets for early intervention.

## Materials and Methods

### Participants

Infants AR were recruited from Eisner Health, Children’s Hospital Los Angeles (Los Angeles, CA, United States), and Ventura County Medical Center (Ventura, CA, United States). These infants were either born before 36 weeks gestation or categorized as high risk based on the California Code of Regulations criteria (Regulations, 2020). This AR group was broadly defined and not strictly standardized to one risk profile, but excluded infants with unstable medical conditions. Infants were considered to be at-risk due to a preponderance of biological risk factors including prematurity, congenital anomalies (e.g., cardiac or brain) necessitating surgical intervention, or a complicated post-natal medical course [including devices to supplement for body functions (i.e., ventilation) or use of supplemental agents (blood products, oxygen, etc)]. See Table 1 for a description of each infant’s health status.

**Table 1:** Description of at risk (AR) participants.

Infant	Gestational age at birth	Pregnancy type	Infant health issues
AR1	34 weeks	Singleton	Respiratory (supplemental oxygen)
AR3	31 weeks	Singleton	Cardiac/respiratory life support, multiple infections, hematologic support (blood transfusions)
AR4	40 weeks	Singleton	Cardiac, Respiratory (mechanical ventilation, supplemental oxygen), vocal cord paralysis
AR5	33 weeks	Singleton	Cardiac (patent ductus arteriosus), Respiratory (pulmonary hyperplasia, supplemental oxygen), hematologic support (blood transfusion)
AR6	34 weeks	Singleton	Gastrointestinal (jaundice, poor feeding)
AR7	33 weeks	Singleton	Cardiac (murmur)
AR8	25 weeks	Multiple (twin)	Cardiac (patent ductus arteriosus), Respiratory (supplemental oxygen), Gastrointestinal (Gastronomy tube, reflux)
AR9	25 weeks	Multiple (twin)	None reported
AR10	30 weeks	Multiple (twin)	Cardiac (Twin-twin transfusion, patent ductus arteriosus), Respiratory (respiratory distress syndrome, supplemental oxygen)
AR11	30 weeks	Multiple (twin)	None reported
AR12	34 weeks	Singleton	Neurologic (hydrocephalus, shunt, encephalocele)

Infants with TD were recruited in-person at Eisner Health (Los Angeles, CA, United States), *via* fliers posted at the University of Southern California (USC), or by word-of-mouth. Infants were from singleton, full-term births. Infants that experienced birth complications, had any known visual, orthopedic or neurologic impairment at the time of testing, or scored at or below the 5th percentile for their age on the Bayley Scales of Infant Development, 3rd edition were excluded.

In total, 11 infants AR and 21 infants with TD participated in the study. The study was approved by the Institutional Review Board of the University of Southern California. A parent or legal guardian signed an informed consent form prior to their infants’ participation.

### Procedures

**Figure 1:**
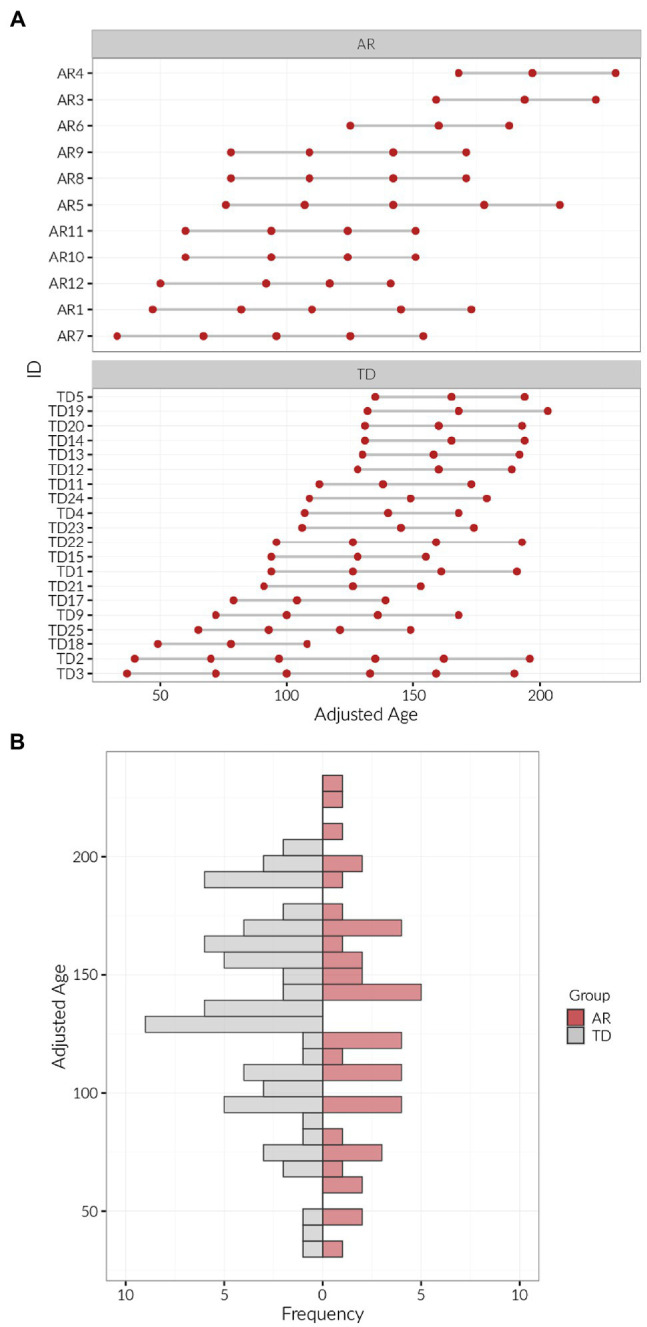
Timepoints and frequency of visits for infants at risk (AR) and with typical development (TD) collected during the development of reaching. **(A)** Each red dot represents a measurement, plotted against adjusted age. **(B)** Distribution of ages of AR and TD groups was noted to be roughly equal.

We assessed infants longitudinally across the time period in which reaching emerges. Age was adjusted for prematurity if appropriate using the equation: adjusted age (in days) = chronological age (in days)—number of days born before due date. We visited the infants in their homes beginning as early as 2 months of age. At this first visit, we collected health history and demographic information (Table 2). Subsequent visits were scheduled once per month and kept time between visits consistent at 1 month +/− 7 days. Our goal was to start data collection before the infants started reaching and continue until they were skilled at reaching (the infant reached for the object with a direct trajectory and pre-shaped hand). Total number of visits ranged from 1 to 6, but most participants (81.3%) had 3 or 4 visits. Across all visits, the age of infants AR ranged from 33 to 230 days old and the age of infants with TD ranged from 37 to 203 days at time of observation. The distribution was noted to be roughly equal for both groups (Figure 1).

**Table 2:** Demographic characteristics of participants (AR = at risk, TD = typical development).

Characteristic	AR, *N* = 11^1^	TD, *N* = 21^1^	*p*-Value^2^
*Gender*			
Female	9 (82%)	12 (57%)	0.2
Male	2 (18%)	9 (43%)	
*Ethnicity*			
Hispanic/Latino	8 (73%)	15 (71%)	>0.9
Not Hispanic/Latino	3 (27%)	6 (29%)	
*Race*			
American Indian/Alaskan Native	3 (27%)	1 (4.7%)	0.07
Asian	1 (9.1%)	0 (0%)	
Black/African	2 (18%)	1 (4.7%)	
Other	2 (18%)	9 (43%)	
White	3 (27%)	10 (48%)	
*Highest education of parent*			
High School	1 (9.1%)	5 (24%)	0.5
Associate	3 (27%)	1 (4.7%)	
Some College	3 (27%)	3 (14%)	
Bachelor	2 (18%)	3 (14%)	
Master	0 (0%)	2 (9.5%)	
Doctorate	1 (9.1%)	5 (24%)	
Declined	1 (9.1%)	2 (9.5%)	

1 Statistics presented: *n* (%).

2 Statistical tests performed: Fisher’s exact test.

Each visit consisted of video recording, electroencephalography, and full-day wearable sensor monitoring of arm movements. The methods and results were previously published for electroencephalography (Hooyman et al., 2018; Xiao et al., 2018) and wearable sensor (Trujillo-Priego et al., 2017; Shida-Tokeshi et al., 2018) full-day arm movement data. In this paper, we focus on video data of reaching toward a toy.

Each visit started with a 5-min video recording of the infant’s spontaneous movement in supine, while they wore a wearable sensor on each arm. The wearable sensors (APDM, Inc., Portland, OR, United States) were inserted into custom arm sleeves and were placed just proximal to the infant’s wrist joints.

This was followed by an assessment of electrical activity of the brain using electroencephalography (EEG). Infants wore a 32-channel EEG cap and sat the lap of their caregiver, who held the infant securely at the trunk. The caregiver provided the necessary level of trunk support for the infant to maintain a stable sitting posture A baseline EEG trial was administered with a spinning globe for 1 min. The globe toy was held out of reach of the infant and was to encourage visual focus and a still head during baseline EEG. Baseline EEG was followed by five 20-s periods where the infant was free to reach for a graspable toy. Each 20-s reaching period was alternated with a 20-s no reaching period where no toy was presented. For each 20-s reaching period, a toy was presented by the researcher at the infant’s midline, shoulder height, and within the infant’s reach. The toy was approximately 11.5 × 11.5 cm, with a spinning rattle in the middle, and a handle for grasping on four different sides. The researcher held the toy at the top or bottom, so the right and left sides of the toy were available for grasping. If the infant successfully grasped the toy, they were allowed to explore it briefly before it was removed from the infant’s grasp and presented again until the 20-s period ended. If the infant dropped the toy in the middle of the reaching period, the toy was then presented again to the infant at midline. Social interaction was present throughout the data collection. Most social interaction occurred between the researcher and the infant as they were facing one another (e.g., when first presenting the toy, the researcher would say something like “Look!” to draw attention to the toy; or when the infant would grasp the toy successfully, the researcher would praise the infant briefly). Caregivers would interact with the infants during short breaks when reaching was not being assessed. One video camera was positioned to record reaching behavior and was placed in front and slightly offset either to the left or right side of the infant, depending on home configuration.

At the end of each visit, we measured the infant’s weight, body and limb lengths, and head and limb circumferences and administered the motor, cognitive, and language scales of the Bayley Scales of Infant and Toddler Development, 3rd edition. The infant’s family received $40 and souvenir photos for each data collection visit.

### Data Analysis

Video data of reaching behavior was behavior-coded by a single coder using ELAN frame-by-frame analysis software (version 4.6.2). A second coder then coded 20% of the video data for reliability. For this portion of the analysis, we identified when each specific reach attempt was made and the outcome of the reach attempt. A reach attempt was coded to begin when the infant directed the arm toward the toy. The infant must have displayed purposeful intention of toy-directed movement through visual fixation either immediately prior to initiating the reach attempt or at any point during the attempt. The attempt ended when the infant either failed to contact the toy (unsuccessful), or the infant contacted the toy (successful). Reach attempts were considered to be bilateral if both hands attempted a reach within 1 s of each other. All other reaches were considered to be unilateral.

#### Reaching Outcomes

The outcome of each reach attempt was defined as follows: miss = a reaching attempt was made but the infant did not contact the toy; touch = a reaching attempt was made and ended in toy contact but not toy acquisition; grasp: [partial] = a reaching attempt was made and ended in toy acquisition using fingers only or part of the hand, such as an ulnar grasp; [whole] = a reaching attempt was made and ended in toy acquisition with a palmar grasp that included both the palm and thumb. Only the most advanced reach outcome was documented; for example, if the infant touched the toy and then grasped it, the outcome was considered to be a grasp. If the infant grasped the toy first but then switched to touching, the outcome was still considered a grasp.

Touching was not distinguished between hitting and touching movements as these are both thought to be pre-grasping behaviors (Rocha et al., 2013). Grasping was divided into partial-hand grasp and whole-hand grasp as transition from the former to the latter could indicate more advanced grasping skill (Barrett et al., 2008). The frequencies of reach outcomes (miss, touch, partial-hand grasp, whole-hand grasp) were totaled for each infant at each visit. Proportion of each reach outcome was calculated by dividing the total frequency of the specific outcome by the total number of reaches for each infant at each visit.

#### Reaching Hand Use

After manual counting of reach attempts and outcomes, we totaled the number of unilateral reaches, which combined both left- and right-unilateral attempts. We also totaled the number of right-handed reaches, including both unilateral and bilateral attempts. Proportion of unilateral attempts was calculated by dividing the number of unilateral reaches by total reaching attempts. Proportion of right hand reaches was calculated by dividing the number of right-handed reaches by total reaching attempts. We chose to present proportion data on unilateral attempts and right-handed attempts in this study, from which one can infer bilateral attempts (attempts were either unilateral or bilateral) and left-handed attempts (attempts were either right-handed or left-handed).

To calculate difference in proportion of successes and whole-hand grasps, we first totaled the frequency of right-hand and left-hand successes (including touch, partial-hand grasp, and whole-hand grasp) and frequency of whole-hand grasps. Proportion was then calculated for the right and left hands’ successes and whole-hand grasps. Difference in proportion of successes and whole-hand grasps were calculated by subtracting the proportion on the left from that on the right to obtain the final value. Positive values would therefore indicate greater proportion of right-sided successes or grasps, and negative values indicate greater proportion of left-sided successes or grasps. A larger absolute value would signify a greater disparity between hands. We excluded all visits that only had no or only one reaching attempt from this difference in hand use analysis.

All data visualization was plotted according to the age of the infant at each visit and the points between visits were connected to allow visualization of patterns across time.

### Statistical Analysis

All statistical analyses were conducted in R (v3.6.3). Demographic characteristics of the sample were computed using the gtsummary package (Sjoberg et al., 2020). We examined longitudinal trends in developmental variables (Bayley subscales), reaching attempts, reaching outcomes, and hand use (proportion of right-handed and unilateral reaching attempts and proportion of hand use differences in successful reaches and whole-hand grasps), over adjusted age. Longitudinal trends were plotted overall using a Lowess smoother for continuous outcomes and a quasibinomial smoother for proportion outcomes, by developmental group, in the ggplot2 package (Wickham, 2016). We analyzed Bayley scores, total reaches, difference in successes, difference in whole-hand grasps, and unilateral reaches using mixed-effects linear regression models with the lmer function in the lme4 package. For reach outcome ratio variables, we used a mixed-effects logistic regression model weighted by total number of reaches, with a distinct model for each reach outcome. These models were performed with the glmer function using a binomial distribution and logit link. All models included a random intercept for participant. We examined the difference in longitudinal trends between groups (AR vs. TD) by testing for an interaction between group and adjusted age, and between group and adjusted age-squared. A significant interaction between group and adjusted age would indicate a difference between groups in the linear component of a variable’s change with increasing adjusted age, while an interaction between group and adjusted age-squared would indicate a difference between groups in the quadratic component of a variable’s change with increasing adjusted age. Non-significant interaction terms (*p* < 0.05) were omitted from the final model, as well as non-significant age-squared terms, and the full model was compared to the reduced model using the likelihood ratio test. We examined residual diagnostics (e.g., residuals vs. leverage) to determine whether any infant was contributing undue influence to the results. Regression models were run using the lme4 package (Bates et al., 2015).

Because the previous literature suggests that decreased variability in movement patterns is a sign of atypical development, we tested for significant differences in within-infant variance between groups for the hand use outcomes (i.e., hand differences in proportion of successful reaches and whole-hand grasps). Outcome values were centered on each infant’s mean to produce intra-individual deviance scores. Then, we used Levene’s test for homogeneity of variances to determine differences between TD vs. AR groups in within-infant variance considering the whole study period (Fox, 2016).

## Results

### Descriptive Variables

All participants (*n* = 11 infants AR and *n* = 21 infants with TD) had at least three assessments over the course of 3 months, except for one participant in the TD group, who had only one assessment. In rare cases, participants had as many as 5 (*n* = 3 TD) or 6 (*n* = 2 AR) assessments over the study period. Timepoints and frequencies of visits are noted in Figure 1. We coded a total of 115 sessions for reaching behavior and 23 sessions (20%) were coded to assure reliability. The inter-rater reliability was 85.2% (Cohen’s *κ* = 0.852; McHugh, 2012) which was greater than our criteria of 80% for reliable coding. Our AR and TD groups had no significant differences in any demographic variables, including sex, race/ethnicity, and parental education (Table 2). We performed an *ad-hoc k*-means cluster analysis to determine if there were any demographic sub-groups within the AR group; however, the identified clusters were defined almost exclusively by gestational age.

**Figure 2:**
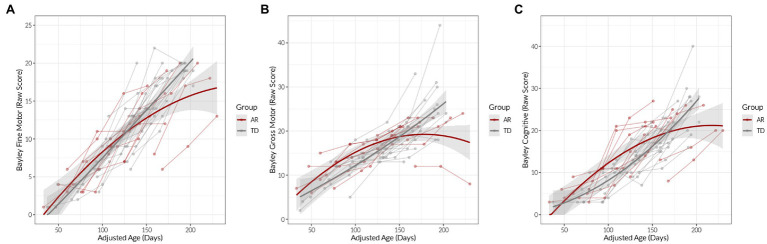
Developmental scores measured over age (adjusted for prematurity as appropriate), for the at risk (AR) and typically developing (TD) groups. Points indicate observed individual values. Lowess-smoothed lines with 95% confidence bands are also shown. **(A)** Bayley fine motor scale (raw score), **(B)** Bayley gross motor scale (raw score), **(C)** Bayley cognitive scale (raw score).

There were differences in the longitudinal trajectories of developmental scores between groups (Figure 2; Table 3A). Bayley fine motor score increased with adjusted age for both groups (*B*_age_ = 1.09, *p* < 0.001), but increased faster for TD infants (B_age × group_ = 0.28, *p* = 0.006). Bayley gross motor score also increased more for the TD group with age and did not exhibit a “leveling-off” effect that the AR group did (B_age × group_ = 0.65, *p* = <0.001; B_age-squared × group_ = 0.05, *p* = 0.049). We did find one AR infant had high residuals and leverage in this model; upon removing this infant from analysis the quadratic effect was no longer significant, but the interaction did still indicate that TD group increased more over time than the AR group. Lastly, we found that both groups increased in their Bayley cognitive scores over time (B_age_ = 1.50, *p* < 0.001) but scores increased faster for TD infants (B_age-square × group_ = 0.08, *p* = 0.01).

**Table 3:** Mixed-effects negative binomial (total reaches) or weighted logistic (all other outcomes) regression on reaching outcomes of several developmental (A) and reaching (B) frequency and laterality variables on adjusted-age (10-day increase), adjusted age-squared, group (AR = at risk, TD = typical development), and interactions.

A										
Predictors	Bayley fine motor (raw score)			Bayley gross motor (raw score)			Bayley cognitive (raw score)			
	Estimates	CI		Estimates	CI		Estimates		CI	
Age	1.087^***^	0.941 to 1.233		0.791^***^	0.572 to 1.009		1.502^***^		1.244 to 1.760	
Group	0.948	−0.884 to 2.781		−1.017	−3.585 to 1.551		−2.374		−5.284 to 0.537	
Age × Group	0.280^**^	0.086 to 0.473		0.652^***^	0.365 to 0.940		0.272		−0.063 to 0.608	
Age^2^				−0.037^*^	−0.072 to −0.002		−0.022		−0.064 to 0.020	
Age^2^ × Group				0.051^*^	0.001 to 0.102		0.080^**^		0.021 to 0.140	
Observations	115			115			115			
Marginal *R*^2^	0.777			0.638			0.715			
**B**										
	**Reaching attempts (frequency)**		**Difference in proportion of successes (R vs. L)**		**Difference in proportion of whole-hand grasps (R vs. L)**		**Unilateral reaches (proportion)**		**Right-sided reaches (proportion)**	
**Predictors**	**Estimates**	**CI**	**Estimates**	**CI**	**Estimates**	**CI**	**Estimates**	**CI**	**Estimates**	**CI**
Age	−2.353	−6.316 to 1.610	−0.061	−0.177 to 0.056	−0.053	−0.180 to 0.073	0.016	−0.069 to 0.101	−0.082	−0.212 to 0.048
Age^2^	0.919^***^	0.441 to 1.397	0.019	−0.002 to 0.040	0.015	−0.007 to 0.038	−0.009	−0.024 to 0.005	−0.004	−0.020 to 0.012
Group	0.271	−0.368 to 0.910	−0.005	−0.034 to 0.024	−0.014	−0.046 to 0.017	−0.003	−0.023 to 0.018	−0.009	−0.031 to 0.013
Age × Group									−0.002	−0.005 to 0.001
Age^2^ × Group									0.005^*^	0.000 to 0.009
Observations	115		94		93		105		105	
Marginal *R*^2^	0.269		0.059		0.029		0.040		0.061	

Non-significant interactions and polynomial terms were excluded from the final model. 95% confidence intervals for parameter estimates are displayed. Results are from 32 participants across 115 visits.

* *p* < 0.05.

** *p* < 0.01.

*** *p* < 0.001.

### Reaching Attempts and Outcomes

**Figure 3:**
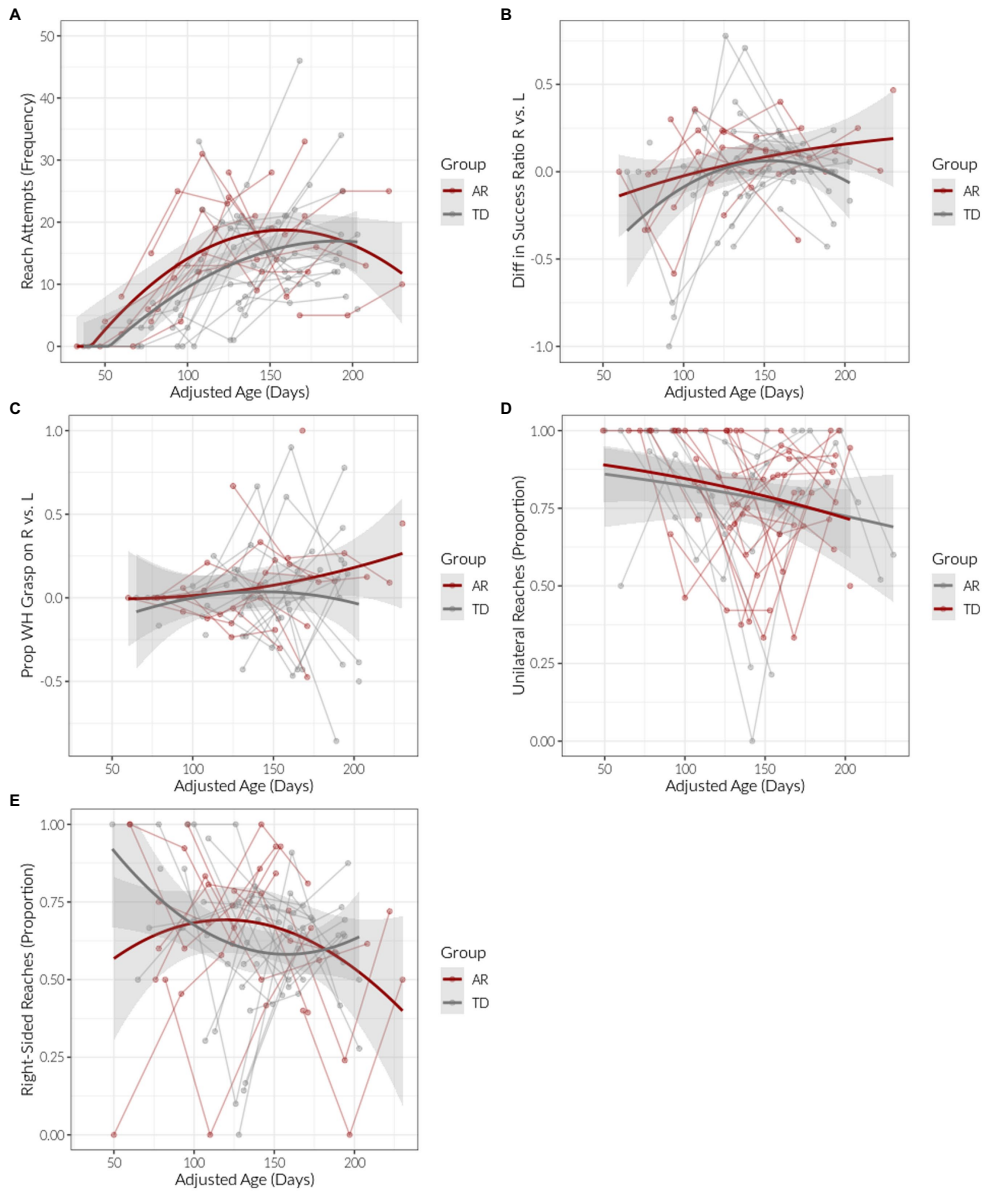
Reaching hand use outcomes by age (adjusted for prematurity as appropriate) for infants at risk (AR) and with typical development (TD). Points indicate observed individual values. Lowess-smoothed lines with 95% confidence bands are also shown. **(A)** Frequency of reach attempts. **(B)** Difference in success ratio for right vs. left hands. **(C)** Proportion of whole hand grasps for right vs. left hands. **(D)** Proportion of unilateral reaches. **(E)** Proportion of right arm reaches.

**Figure 4:**
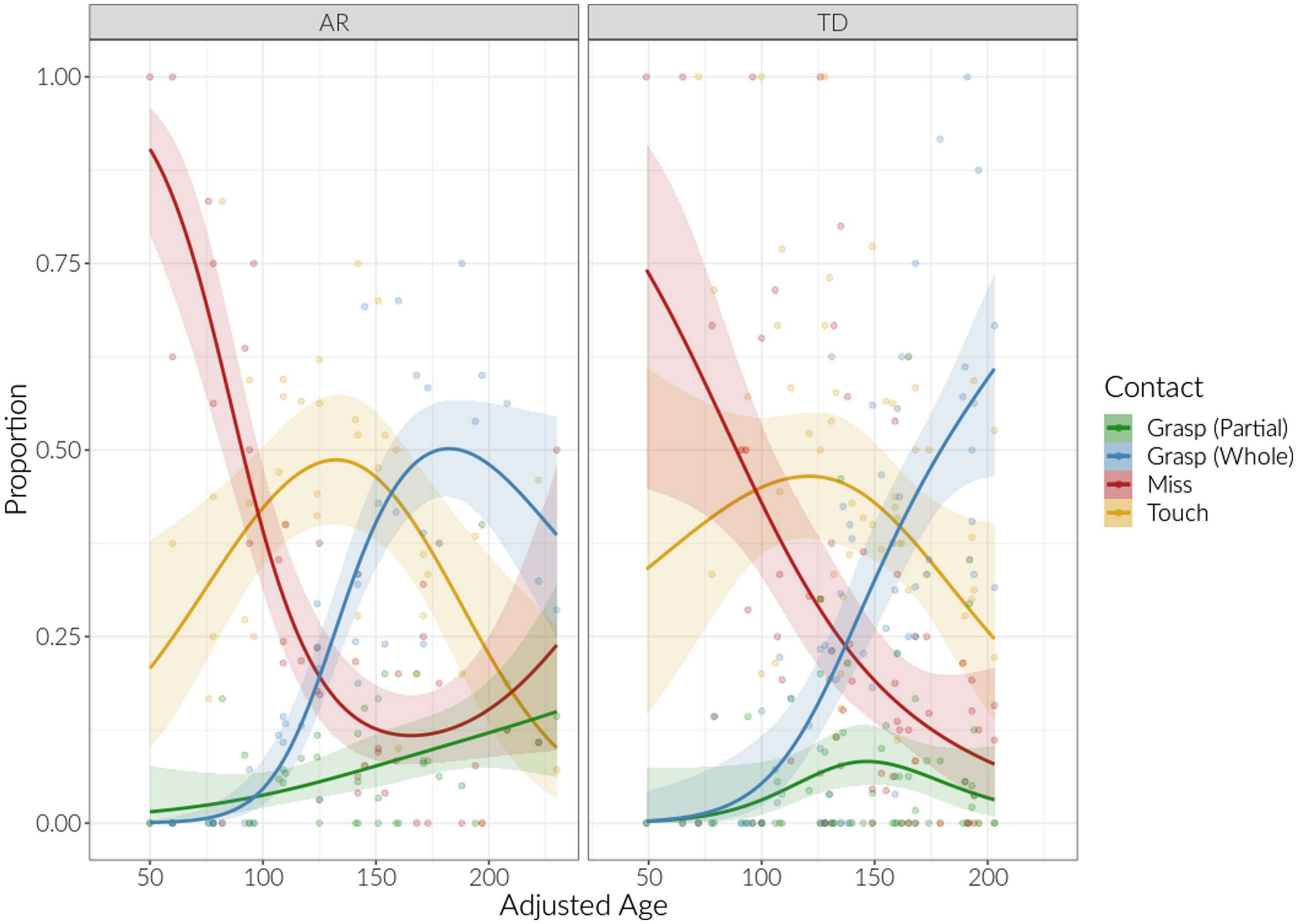
Proportion of reaching outcomes by age (adjusted for prematurity as appropriate) and group (AR = at risk, TD = typical development). Displayed are individual points and quasibinomial-smoothed line, with 95% confidence bands.

Reaching frequency increased consistently over age (adjusted for prematurity as appropriate) for both groups (B_age_ = 0.919, *p* < 0.001, Figure 3A). The longitudinal pattern of reach outcomes was largely similar between both groups (Table 4; Figure 4). Mixed-effects logistic regression models, weighted by total reaches, showed that the proportion of missed reaches decreased over adjusted age for both groups (B_age_ = −0.20, *p* < 0.001), but then subsequently increased for the AR group, with a lower proportion of misses for the TD group (B_age-squared_ = 0.03, *p* < 0.001; B_age-squared × group_ = −0.02, *p* = 0.03). The proportion of reaches with a touch outcome increased with age initially then decreased (B_age-squared_ = −0.01, *p* = 0.002). The proportion of partial grasps had no significant change over time for both groups (B_age_ = 0.10, *p* = 0.08), whereas the proportion of whole-hand grasps increased over time for both groups (B_age_ = 0.40, *p* < 0.001; B_age-squared_ = −0.04, *p* < 0.001).

**Table 4:** Mixed-effects weighted logistic regression on four reaching outcomes on adjusted-age (10-day increase), adjusted age-squared, group (at risk or typical development), and interactions.

Predictors	Miss		Touch		Grasp Partial		Grasp Whole	
	Log-Odds	CI	Log-Odds	CI	Log-Odds	CI	Log-Odds	CI
Age	−0.20 ^***^	−0.26 to −0.14	−0.03	−0.08 to 0.01	0.10	−0.01 to 0.22	0.40^***^	0.29 to 0.51
Group	0.30	−0.27 to 0.88	−0.04	−0.37 to 0.29	0.25	−0.40 to 0.89	−0.20	−0.70 to 0.29
Age^2^	0.03^***^	0.02 to 0.04	−0.01^**^	−0.02 to −0.01	−0.00	−0.02 to 0.02	−0.04^***^	−0.05 to −0.02
Age × Group	−0.06	−0.15 to 0.04	−0.00	−0.08 to 0.07	−0.01	−0.20 to 0.18	−0.03	−0.19 to 0.12
Age^2^ × Group	−0.02^*^	−0.04 to −0.00	0.00	−0.01 to 0.02	−0.03	−0.06 to 0.01	0.02	−0.01 to 0.04
Observations	105		105		105		105	
*R*^2^ Tjur	0.073		0.088		0.005		0.032	

* *p* < 0.05.

** *p* < 0.01.

*** *p* < 0.001.

### Hand Use

There were no age or group effects on the mean difference in proportion of successes (Figure 3B; Table 3B) or whole-hand grasps for the left vs. right hand (Figure 3C; Table 3B). There were also no age or group effects for unilateral reaches (Figure 3D). The AR group did not change the use of their right side over time, while the TD group showed evidence of more right-hand use at younger ages, followed by decreasing use, with subsequent increased use at higher age (B_age-squared × group_ = 0.005, *p* = 0.04; Figure 3E; Table 3B). When considering the figure and statistical results for proportion of right-hand use over time, it is important to note that the raw data are shown in Figure 3E but the statistical model (mixed effect model) centers all of the values on each participant’s mean value.

Though the mean difference in proportion of whole-hand grasps for the left vs. right hand did not differ between groups, Levene’s test did show that there was overall less intra-individual variation in this variable for the AR group compared to the TD group (*F* = 4.24, *p* = 0.04). There was no evidence that the intra-individual variation in difference of successful reaches between hands differed between groups (*F* = 0.14, *p* = 0.71).

## Discussion

The goal of this study was to investigate the outcomes and hand use of reaching attempts as infants AR and with TD gained reaching skill. Overall, our results showed differences in proportion of missed reaches, proportion of right-hand use over time, and intra-individual variance in hand use for grasping between groups. Across the time period in which they learned to reach, infants AR had a higher proportion of missed reaches, more consistent proportion of right-hand use, and less intra-individual variance in hand use for grasping compared to infants with TD.

### Descriptive Variables

First, to describe the developmental trajectories of the two groups, we assessed Bayley gross motor, fine motor, and cognitive scores. Bayley fine motor and cognitive scores increased with age for both groups, but increased more rapidly for infants with TD. Bayley gross motor scores increased in the TD group but demonstrated a “plateau” effect for the AR group after approximately 150 days of adjusted age. These findings are consistent with other studies that demonstrate decreased rate of change in developmental trajectories in at-risk populations as a whole (Durrant et al., 2020), although it is very possible that individual infants would have highly variable developmental trajectories (Su et al., 2017).

### Reaching Outcomes

Reaching frequency tended to increase then plateau in both groups. However, group differences were significant when considering reaching outcomes: proportion of missed reaches declined with age in both groups but then increased after 160 days in the AR group in a “rebound” effect. Touches were not significantly different between groups. Lastly, both groups had increased proportion of whole-hand grasping with age and did not show group differences.

These findings suggest that the proportion of reaches resulting in a touch or grasp do not present very differently over time in the AR and TD groups. In fact, we have seen similar changes over time in reach frequency (Goncalves et al., 2013; Nogueira et al., 2015), reach success (Heathcock et al., 2008; Chen et al., 2015) and touching and grasping (de Almeida Soares et al., 2014) in other AR and TD groups. It is only when considering proportion of missed reaches, which had previously not been done, do we see subtle group differences. Proportion of missed reaches was significantly higher in the AR group than the TD group. This may have implications for continued motor skill development.

### Hand Use

Both groups tended to have the same proportion of unilateral reaches throughout the study period. Right-handed reaches exhibited a quadratic effect for the TD group; on average infants with TD tended to reach more with the right hand at earlier ages, then decreased proportion of right-hand use to near 50% by 150 days of age, then appeared to return to their original proportion of right-hand use by 200 days of age. In contrast, those in the AR group on average had right-hand use close to 60% across the whole period of observation. Other literature on early infant hand use similarly indicate that infants with TD have fluctuations in hand preference during reaching and do not show a stable preference across the pre-reaching period (Lynch et al., 2008) or the first year of life (Corbetta and Thelen, 1999). From a theoretical perspective, infants with TD appear to change how they reach over time and prefer to reach with one arm or the other for short periods, and infants AR may not show these short periods of preference. This should be examined more closely in future studies, preferably linked to neuroimaging and developmental outcomes.

In our study, we also found a significantly lower intra-individual variance in our AR group with left- and right-hand use with whole-hand grasping. This finding was not significant when including both touching and grasping. The theoretical importance of movement variability was previously discussed by Thelen and Smith: in 1994, they proposed that infants will increase motor variability to explore new learning opportunities (Thelen and Smith, 1994). In addition, Corbetta et al. (2018) proposed that infants learning to reach for objects will increase movement variability, arising from exploration of different types of behavior (Corbetta et al., 2018). Infants will then select and fine-tune over time the movement pattern that best accomplishes the goal, with a corresponding decrease in movement variability. We are the first to quantitatively measure movement variability in unilateral vs. bilateral and right vs. left reaching attempts within and across assessments longitudinally during the emergence of reaching. Lower variance when grasping with different hands in the AR group may suggest that these infants have less exploration when successfully grasping objects; instead, they grasp the same way using the left or right hand consistently over visits. Since grasping is more difficult than simply contacting a toy with the hand, this may suggest that infants AR demonstrate less exploration when performing higher-level skills or tasks.

Future directions for identifying typical and atypical development include a closer look into missed reaches, movement variability, and individual differences. What were the movement characteristics of the reaches that were missed, and how do they compare to ones that were successful? In other words, can we understand why the infants in the AR group tend to miss their reaches more than infants in the TD group, and why? Also, use of other quantitative measures of movement variability will be an important next step in understanding how infants at risk have less variable movement patterns while learning to reach. Lastly, since the AR group is broadly defined, a closer look into the individual movement pattern differences may be helpful to distinguish those who may go on to develop developmental disabilities. Our study adds insight into how reaching outcomes and hand use may be different in infants AR who are learning to reach. From here, we can focus on early identification of individual infants that may exhibit these differences.

### Limitations

We have limitations to consider when interpreting the results of this study. Namely, we do not know the final developmental outcomes of the AR group, roughly half of whom are expected to receive diagnoses consistent with developmental disabilities (e.g., cerebral palsy, dyspraxia, and attention-deficit hyperactivity disorder) while others will not.

Methodologically, we recognize some limitations to our video coding and variable selection. Video coding of missed reaches are subjective as they require the coder to infer the intention of the infant. Reliability coding by a second coder increases confidence in this variable, but certainly does not eliminate error. With variable selection, we chose to assess proportion of unilateral and right-hand reaches. However, at some visits, especially those at younger ages, the infant reached only a few times during the 5 × 20-s periods, and thus, analyzing only a few reaches may have produced more extreme proportion values at some visits. As our intention was to move beyond studying skilled reaching and study the earlier development of the skill, when infants produce fewer reach attempts and miss the toy on some attempts, we saw these limitations as inherent to our study design. Future studies may use other methods or study designs to further the investigation of reaching skill development.

## Conclusion

In our study of infants AR and with TD as they learned to reach, we found differences between groups in unsuccessful reaches, proportion of right-hand reaches, and grasping variability. Specifically, the infants AR tended to have higher proportions of missed reaches compared to infants with TD. Infants AR also showed a more consistent proportion of right-hand reaches. While infants with TD showed a higher, then lower, then higher again proportion of right-hand reaches infants AR showed a steadier proportion of right-hand reaches across time. Infants AR significantly lower intra-individual variance in the amounts of right- and left-hand use for whole-hand grasping. There was not a group difference in the amounts of right- and left-hand use when considering both touching and grasping. From this, we see that infants AR grasped the toy using the left or right hand more consistently across visits, while infants with TD switched more between using the right or left hand for grasping across visits. Now that we know that infants AR tend to miss more reaches than infants with TD, the next step is to assess how, kinematically, their missed reaches are different. Lastly, because infants AR have more stable use of the right hand for reaching and less individual variance in hand use when grasping compared to infants with TD, we are interested in studying more about the role of variability and “adaptability” in motor skill learning using other quantitative measures of variability.

## Data Availability Statement

The raw data supporting the conclusions of this article will be made available by the authors, without undue reservation.

## Ethics Statement

The studies involving human participants were reviewed and approved by University of Southern California IRB. Written informed consent to participate in this study was provided by the participants’ parent or legal guardian.

## Author Contributions

JZ: conceptualization, methodology, writing—original draft, and visualization. NR: writing—revisions, review, and editing. JS-T: investigation and writing—review and editing. TP: formal analysis, writing—original draft, and writing—review and editing. DV: conceptualization, resources, and writing—review and editing. BS: conceptualization, methodology, supervision, funding acquisition, and writing—review and editing. All authors contributed to the article and approved the submitted version.

## Funding

This work was supported by a grant from the Bill & Melinda Gates Foundation (OPP1119189; PI: BS). This work was also supported by grants UL1TR001855 and UL1TR000130 from the National Center for Advancing Translational Science (NCATS) of the U.S. National Institutes of Health. The content is solely the responsibility of the authors and does not necessarily represent the official views of the National Institutes of Health.

## Conflict of Interest

The authors declare that the research was conducted in the absence of any commercial or financial relationships that could be construed as a potential conflict of interest.

## Publisher’s Note

All claims expressed in this article are solely those of the authors and do not necessarily represent those of their affiliated organizations, or those of the publisher, the editors and the reviewers. Any product that may be evaluated in this article, or claim that may be made by its manufacturer, is not guaranteed or endorsed by the publisher.

## References

[ref1] Barrett T. M. Traupman E. Needham A. (2008). Infants' visual anticipation of object structure in grasp planning. Infant Behav. Dev. 31, 1–9. doi: 10.1016/j.infbeh.2007.05.004, PMID: 17624439

[ref2] Bates D. Maechler M. Bolker B. Walker S. (2015). Fitting linear mixed-effects models using lme4. J. Stat. Softw. 67, 1–48. doi: 10.18637/jss.v067.i01

[ref001] Boxum A. G. La Bastide-Van Gemert S. Dijkstra L.-J. (2017). Development of the quality of reaching in infants diagnosed with cerebral palsy: a kinematic study. Dev. Med. Child Neurol. 59, 1164–1173. 28877349 10.1111/dmcn.13538

[ref3] Bhat A. N. Galloway J. C. (2006). Toy-oriented changes during early arm movements: hand kinematics. Infant Behav. Dev. 29, 358–372. doi: 10.1016/j.infbeh.2006.01.005, PMID: 17138291

[ref4] Chen C. Y. Tafone S. Lo W. Heathcock J. C. (2015). Perinatal stroke causes abnormal trajectory and laterality in reaching during early infancy. Res. Dev. Disabil. 38, 301–308. doi: 10.1016/j.ridd.2014.11.014, PMID: 25577180

[ref5] Corbetta D. DiMercurio A. Wiener R. F. Connell J. P. Clark M. (2018). How perception and action fosters exploration and selection in infant skill acquisition. Adv. Child Dev. Behav. 55, 1–29. doi: 10.1016/bs.acdb.2018.04.001, PMID: 30031432

[ref6] Corbetta D. Thelen E. (1999). Lateral biases and fluctuations in infants’ spontaneous arm movements and reaching. Dev. Psychobiol. 34, 237–255. doi: 10.1002/(SICI)1098-2302(199905)34:2<237::AID-DEV1>3.0.CO;2-#, PMID: 10331149

[ref7] de Almeida Soares D. Cunha A. B. Tudella E. (2014). Differences between late preterm and full-term infants: comparing effects of a short bout of practice on early reaching behavior. Res. Dev. Disabil. 35, 3096–3107. doi: 10.1016/j.ridd.2014.07.041 25134076

[ref003] de Campos A. C. da Costa C. S. Savelsbergh G. J. P. (2013). Infants with Down syndrome and their interactions with objects: Development of exploratory actions after reaching onset. Res. Dev. Disabil. 35, 1906–1916. 10.1016/j.ridd.2013.03.001 23584171

[ref8] Durrant C. Wong H. S. Cole T. J. Hutchon B. Collier L. Wright A.. (2020). Developmental trajectories of infants born at less than 30 weeks' gestation on the Bayley-III scales. Arch. Dis. Child. Fetal Neonatal Ed. 105, 623–627. doi: 10.1136/archdischild-2019-317810, PMID: 32366516

[ref9] Dusing S. C. Izzo T. A. Thacker L. R. Galloway J. C. (2014). Postural complexity differs between infant born full term and preterm during the development of early behaviors. Early Hum. Dev. 90, 149–156. doi: 10.1016/j.earlhumdev.2014.01.006, PMID: 24485170 PMC3950939

[ref11] Fallang B. Saugstad O. D. Grogaard J. Hadders-Algra M. (2003). Kinematic quality of reaching movements in preterm infants. Pediatr. Res. 53, 836–842. doi: 10.1203/01.PDR.0000058925.94994.BC, PMID: 12612201

[ref12] Fox J. (2016). Applied Regression Analysis and Generalized Linear Models. 3rd Edn. London: Sage.

[ref13] Goncalves R. V. Figueiredo E. M. Mourao C. B. Colosimo E. A. Fonseca S. T. Mancini M. C. (2013). Development of infant reaching behaviors: kinematic changes in touching and hitting. Infant Behav. Dev. 36, 825–832. doi: 10.1016/j.infbeh.2013.09.009, PMID: 24140840

[ref15] Heathcock J. C. Lobo M. Galloway J. C. (2008). Movement training advances the emergence of reaching in infants born at less than 33 weeks of gestational age: a randomized clinical trial. Phys. Ther. 88, 310–322. doi: 10.2522/ptj.20070145, PMID: 18096650

[ref16] Hooyman A. Kayekjian D. Xiao R. Jiang C. Vanderbilt D. L. Smith B. A. (2018). Relationships between variance in electroencephalography relative power and developmental status in infants with typical development and at risk for developmental disability: an observational study. Gates Open Res. 2:47. doi: 10.12688/gatesopenres.12868.2, PMID: 30569037 PMC6266744

[ref17] Lynch A. Lee H. M. Bhat A. Galloway J. C. (2008). No stable arm preference during the pre-reaching period: a comparison of right and left hand kinematics with and without a toy present. Dev. Psychobiol. 50, 390–398. doi: 10.1002/dev.20297, PMID: 18393280

[ref18] Mazzarella J. McNally M. Chaudhari A. M. W. Pan X. Heathcock J. C. (2020). Differences in coordination and timing of pre-reaching upper extremity movements may be an indicator of cerebral palsy in infants with stroke: a preliminary investigation. Clin. Biomech. 73, 181–188. doi: 10.1016/j.clinbiomech.2019.12.024, PMID: 32007826

[ref19] McHugh M. L. (2012). Interrater reliability: the kappa statistic. Biochem. Med. 22, 276–282. PMC3900052 23092060

[ref20] Michel G. F. Babik I. Nelson E. L. Campbell J. M. Marcinowski E. C. (2013). How the development of handedness could contribute to development of language. Dev. Psychobiol. 55, 608–620. doi: 10.1002/dev.21121, PMID: 23754687 PMC4040107

[ref21] Michel G. F. Campbell J. M. Marcinowki E. C. Nelson E. L. Babik I. (2016). Infant hand preference and the development of cognitive abilities. Front. Psychol. 7:410. doi: 10.3389/fpsyg.2016.00410, PMID: 27047431 PMC4803747

[ref23] Nogueira S. F. Figueiredo E. M. Gonçalves R. V. Mancini M. C. (2015). Relation between hand function and gross motor function in full term infants aged 4 to 8 months. Braz. J. Phys. Ther. 19, 52–60. doi: 10.1590/bjpt-rbf.2014.0070, PMID: 25714437 PMC4351608

[ref24] Novak I. Morgan C. (2019). High-risk follow-up: early intervention and rehabilitation. Handb. Clin. Neurol. 162, 483–510. doi: 10.1016/B978-0-444-64029-1.00023-0 31324326

[ref002] Ouss L. Le Normand M.-T. Bailly K. (2018). Developmental trajectorries of hand movemnets in typical infants and those at risk of developmental disroders: an observational study of kinematics during the first year of life. Front. Psychol. 9, 1–15., PMID: 29515472 10.3389/fpsyg.2018.00083 PMC5826068

[ref26] Raju T. N. K. (2012). “The high-risk infant,” in Textbook of Clinical Pediatrics. eds. Elzouki A. Y. Harfi H. A. Nazer H. M. Stapleton F. B. Oh W. Whitley R. J. (Berlin, Heidelberg: Springer), 177–186.

[ref28] Regulations C. C. O. (2020). Eligibility Criteria: Established Risk and High Risk for Developmental Delay.

[ref29] Rocha N. A. de Campos A. C. Silva F. P. Tudella E. (2013). Adaptive actions of young infants in the task of reaching for objects. Dev. Psychobiol. 55, 275–282. doi: 10.1002/dev.21026, PMID: 22539262

[ref30] Ronnqvist L. Domellof E. (2006). Quantitative assessment of right and left reaching movements in infants: a longitudinal study from 6 to 36 months. Dev. Psychobiol. 48, 444–459. doi: 10.1002/dev.20160, PMID: 16886181

[ref31] Sacrey L. R. Whishaw I. Q. (2010). Development of collection precedes target reaching: resting shapes of the hands and digits in 1-6-month-old human infants. Behav. Brain Res. 214, 125–129. doi: 10.1016/j.bbr.2010.04.052, PMID: 20451560

[ref32] Shida-Tokeshi J. Lane C. J. Trujillo-Priego I. A. Deng W. Vanderbilt D. L. Loeb G. E.. (2018). Relationships between full-day arm movement characteristics and developmental status in infants with typical development as they learn to reach: an observational study. Gates Open Res. 2:17. doi: 10.12688/gatesopenres.12813.2, PMID: 29708221 PMC5915838

[ref33] Sjoberg D. D. Hannum M. Whiting K. Zabor E. C. (2020). gtsummary: presentation-ready data summary and analytic result tables. Available at: https://github.com/ddsjoberg/gtsummary, http://www.danieldsjoberg.com/gtsummary/ (Accessed May 13, 2022).

[ref34] Su Y. H. Jeng S. F. Hsieh W. S. Tu Y. K. Wu Y. T. Chen L. C. (2017). Gross motor trajectories during the first year of life for preterm infants with very low birth weight. Phys. Ther. 97, 365–373. doi: 10.1093/ptj/pzx007, PMID: 28339607

[ref35] Thelen E. Corbetta D. Kamm K. Spencer J. P. Schneider K. Zernicke R. F. (1993). The transition to reaching: mapping intention and intrinsic dynamics. Child Dev. 64, 1058–1098. doi: 10.2307/1131327, PMID: 8404257

[ref36] Thelen E. Smith L. B. (1994). A Dynamic Systems Approach to the Development of Cognition and Action. Cambridge, MA: MITPress.

[ref37] Toledo A. M. Tudella E. (2008). The development of reaching behavior in low-risk preterm infants. Infant Behav. Dev. 31, 398–407. doi: 10.1016/j.infbeh.2007.12.006, PMID: 18289691

[ref38] Trujillo-Priego I. A. Lane C. J. Vanderbilt D. L. Deng W. Loeb G. E. Shida-Tokeshi J.. (2017). Development of a wearable sensor algorithm to detect the quantity and kinematic characteristics of infant arm movement bouts produced across a full day in the natural environment. Technologies 5:39. doi: 10.3390/technologies5030039, PMID: 28824853 PMC5558826

[ref39] von Hofsten C. V. (1991). Structuring of early reaching movements: a longitudinal study. J. Mot. Behav. 23, 280–292. doi: 10.1080/00222895.1991.9942039, PMID: 14766510

[ref40] Wickham H. (2016). ggplot2: Elegant Graphics for Data Analysis. New York: Springer-Verlag.

[ref41] Wu H. G. Miyamoto Y. R. Gonzalez Castro L. N. Olveczky B. P. Smith M. A. (2014). Temporal structure of motor variability is dynamically regulated and predicts motor learning ability. Nat. Neurosci. 17, 312–321. doi: 10.1038/nn.3616, PMID: 24413700 PMC4442489

[ref42] Xiao R. Shida-Tokeshi J. Vanderbilt D. L. Smith B. A. (2018). Electroencephalography power and coherence changes with age and motor skill development across the first half year of life. PLoS One 13:e0190276. doi: 10.1371/journal.pone.0190276, PMID: 29329316 PMC5766131

